# Identification of foam cell biomarkers by microarray analysis

**DOI:** 10.1186/s12872-020-01495-0

**Published:** 2020-05-06

**Authors:** Zikai Song, Shijie Lv, Haidi Wu, Ling Qin, Hongyan Cao, Bo Zhang, Shuping Ren

**Affiliations:** 1 Department of Cardiology, The First Hospital of Jilin University, Changchun, Jilin Province China; 2 Department of Orthopedics, Jilin Province FAW General Hospital, Changchun, Jilin Province China; 3 Department of Pediatric Neurology, The First Hospital of Jilin University, Jilin University, Changchun, Jilin Province China; 4 Department of Occupational and Environmental Health, School of Public Health, Jilin University, Changchun, Jilin Province China

**Keywords:** Macrophages, Foam cell, Differential expression gene, GO enrichment, KEGG pathway analysis, Protein interaction network

## Abstract

**Background:**

Lipid infiltration and inflammatory response run through the occurrence of atherosclerosis. Differentiation into macrophages and foam cell formation are the key steps of AS. Aim of this study was that the differential gene expression between foam cells and macrophages was analyzed to search the key links of foam cell generation, so as to explore the pathogenesis of atherosclerosis and provide targets for the early screening and prevention of coronary artery disease (CAD).

**Methods:**

The gene expression profiles of GSE9874 were downloaded from Gene Expression Omnibus (https://www.ncbi.nlm.nih.gov/geo/query/acc.cgi?acc=GSE9874) on GPL96 [HG-U133A] Affymetrix Human Genome U133. A total of 22,383 genes were analyzed for differentially expression genes (DEGs) by Bayes package. GO enrichment analysis and KEGG pathway analysis for DEGs were performed using KOBAS 3.0 software (Peking University, Beijing, China). STRING software (STRING 10.0; European Molecular Biology Laboratory, Heidelberg, Germany) was used to analyze the protein-protein interaction (PPI) of DEGs.

**Results:**

A total of 167 DEGs between macrophages and foam cells were identified. Compared with macrophages, 102 genes were significantly upregulated and 65 genes were significantly downregulated (*P* < 0.01, fold-change > 1) in foam cells. DEGs were mainly enrich in ‘sterol biosynthetic and metabolic process’, ‘cholesterol metabolic and biosynthetic process’ by GO enrichment analysis. The results of KEGG pathway analysis showed all differential genes are involved in biological processes through 143 KEGG pathways. A PPI network of the DEGs was constructed and 10 outstanding genes of the PPI network was identified by using Cytoscape, which include HMGCR, SREBF2, LDLR, HMGCS1, FDFT1, LPL, DHCR24, SQLE, ABCA1 and FDPS. Conclusion: Lipid metabolism related genes and molecular pathways were the key to the transformation of macrophages into foam cells. Therefore, lipid metabolism disorder is the key to turn macrophages into foam cells, which plays a major role in CAD.

## Background

With the development of global economy, metabolic diseases such as hypertension, diabetes and obesity increase, leading to coronary artery disease (CAD) is still one of the major diseases threatening human’s health in this century. In particular, it is worth noticing that although the diagnosis and treatment of CAD are in great development, the incidence of CAD and the trend of youth are still unavoidable, further bring a huge economic and psychological burden to human beings.

Atherosclerotic plaque accumulation in the epicardial arteries is the main pathological mechanism of CAD [1]. Lipid infiltration and inflammatory response run through the occurrence of atherosclerosis (AS) [2]. Endothelial cell dysfunction, expression of cellular adhesion molecules, lipid retention, monocyte recruitment and differentiation into macrophages, foam cell formation, proteolysis, apoptosis, angiogenesis are the key steps of AS [3]. Each of these mechanisms and potential diagnostic and therapeutic targets have been extensively studied. However, the mechanism of CAD has not been fully elucidated.

The data of gene expression profiles have been increased rapidly in recent years, and bioinformatics is widely used to analyze a large number of gene expression profile data to provide new sights for revealing the pathogenesis of CAD, and theoretical basis for early diagnosis, prevention and treatment target selection of CAD [4].

Because foam cells are the characteristic pathological cells of AS. They can be used to find the underlying mechanisms of CAD by detecting the differentially expressed genes. In this study, the differential gene expression between foam cells and macrophages was analyzed to search the key links of foam cell generation, so as to explore the pathogenesis of atherosclerosis and provide targets for the early screening and prevention of CAD.

## Methods

### Microarray data

The gene expression profiles of GSE9874 were downloaded from Gene Expression Omnibus (GEO) (https://www.ncbi.nlm.nih.gov/geo/query/acc.cgi?acc=GSE9874). GSE9874 was performed on GPL96 [HG-U133A] Affymetrix Human Genome U133. The GSE9874 data set contained 60 samples, including 15 non-AS-macrophage samples from subjects without AS, 15 AS-macrophage samples from atherosclerotic tissues, 15 non-AS-foam cells samples from subjects without AS and 15 AS-foam cells samples from atherosclerotic tissues. Macrophages were obtained from human white blood cells of fifteen subjects with atherosclerosis/family history of CAD and from fifteen subjects (sex and age matched) without atherosclerosis/family history of CAD. After collection, all monocyte-derived macrophages from peripheral blood were cultured in absence or presence (foam cells) of ox-LDL from all subjects (healthy and atherosclerotic).

### Principal component analysis (PCA)

**Fig. 1:**
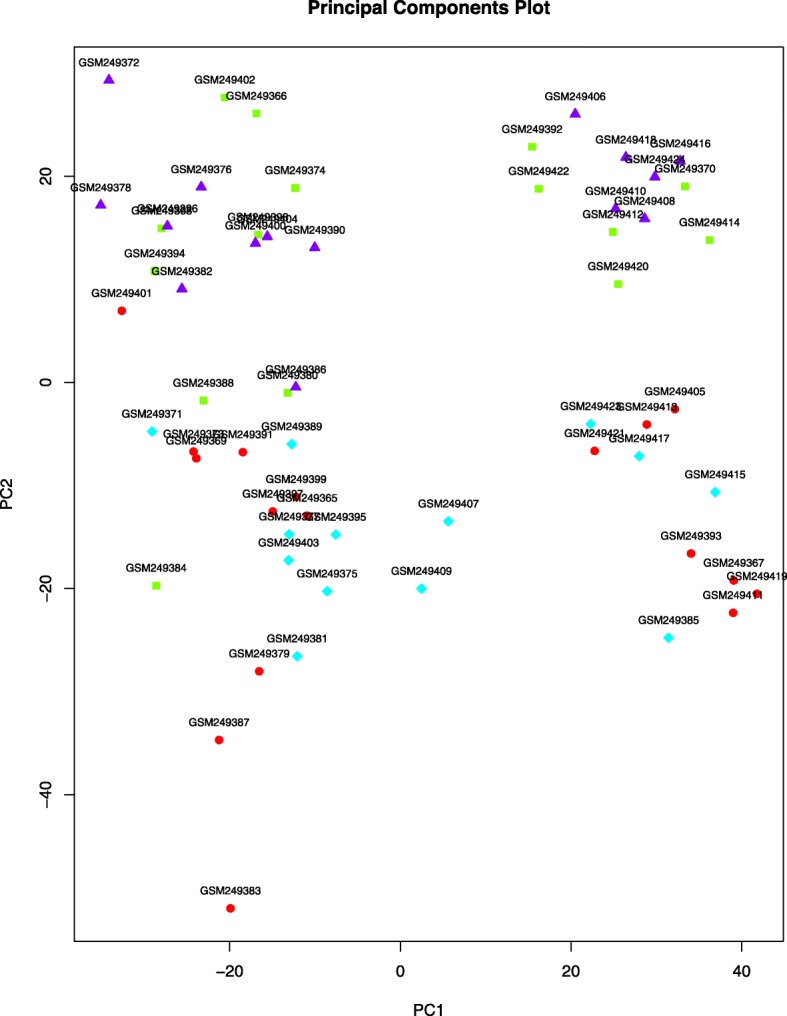
Principal component analysis in four groups

The processed data were downloaded using R package GEO query. The mRNA expression levels of targeted patients and controls were extracted from all the samples and were transformed into log2 scale before further analysis. PCA was performed, and the results were shown in Fig. 1. It was difficult to distinguish gene expression in each group.

**Fig. 2:**
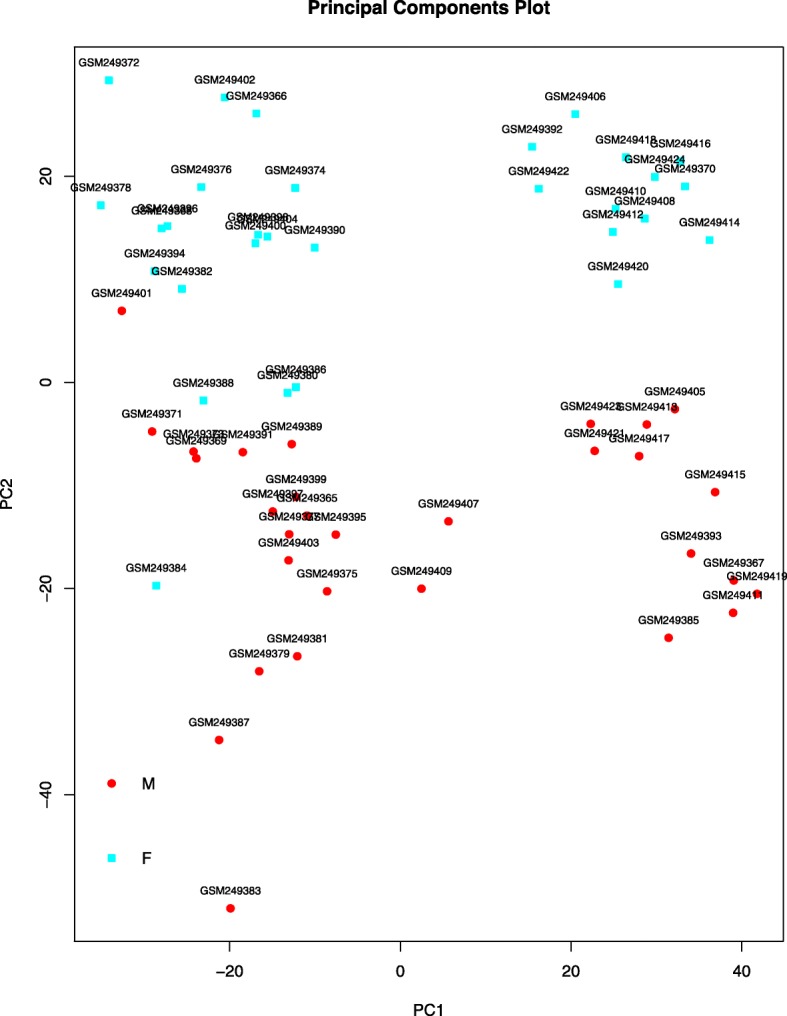
Principal component analysis in two groups

According to the results in Fig. 1, 60 samples were divided into two groups: 30 foam cells samples and 30 macrophages samples. The results of PCA were shown in Fig. 2, which could better distinguish the gene expression of each group.

### Identification of differentially expression genes (DEGs)

A total of 22,383 genes were analyzed for DEGs by Bayes package.

### Gene ontology (GO) enrichment analysis and KEGG pathway analysis

GO enrichment analysis and KEGG pathway analysis for differentially expressed gene were performed using KOBAS 3.0 software (Peking University, Beijing, China), which can be accessed at https://kobas.cbi.pku.edu.cn.

### Protein interaction network analysis

STRING software (STRING 10.0; European Molecular Biology Laboratory, Heidelberg, Germany) was used to analyze the protein-protein interaction (PPI) of differentially expressed genes. PPI refers to the forming of protein complex by two or more protein molecules through non-covalent bonds. STRING can be accessed at https://string-db.org/.

## Results

### Screening of differentially expressed genes

**Fig. 3:**
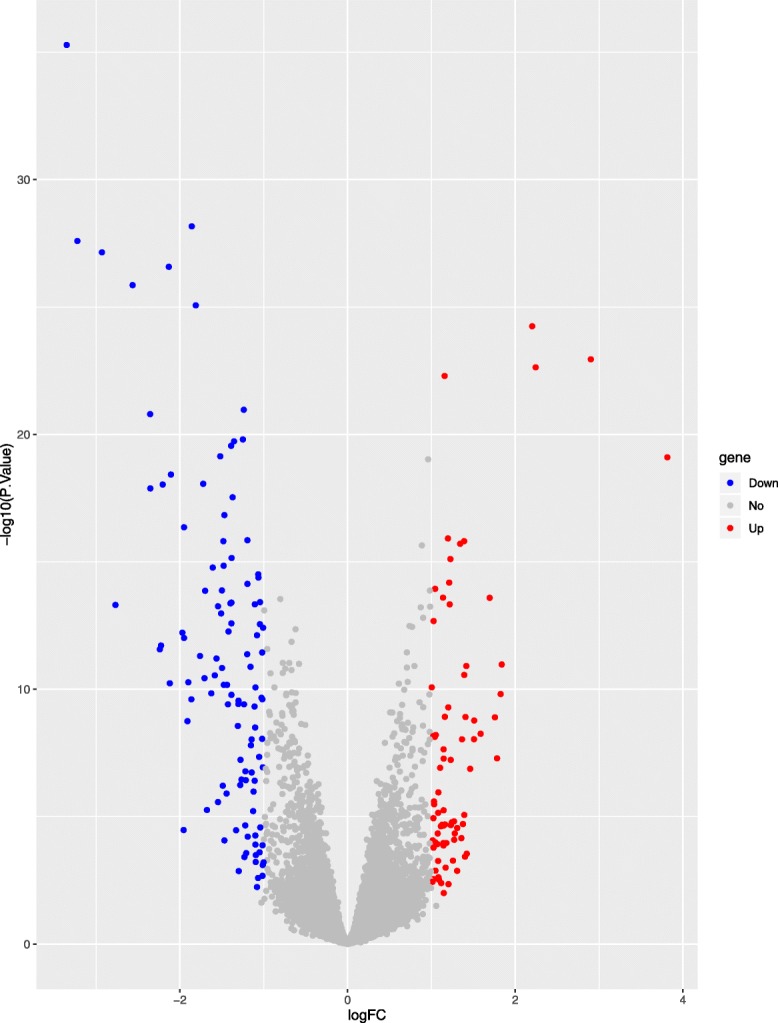
Volcano plots presenting the differences between two groups

**Fig. 4:**
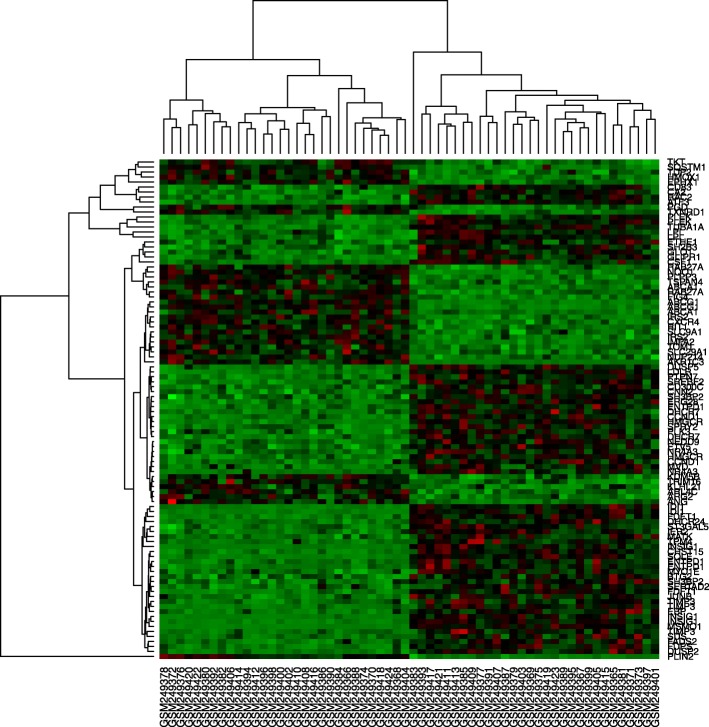
The heatmap of the first 100 differentially expressed genes in two groups

A total of 167 differentially expressed genes between macrophages and foam cells were identified from gene chip GSE9874. Compared with macrophages, 102 genes were significantly upregulated and 65 genes were significantly downregulated (*P* < 0.01, fold-change > 1) in foam cells, and which were plotted in the form of volcano plots (Fig. 3). The top 100 genes were listed in heatmap (Fig. 4).

### GO enrichment analysis and KEGG pathway analysis

**Fig. 5:**
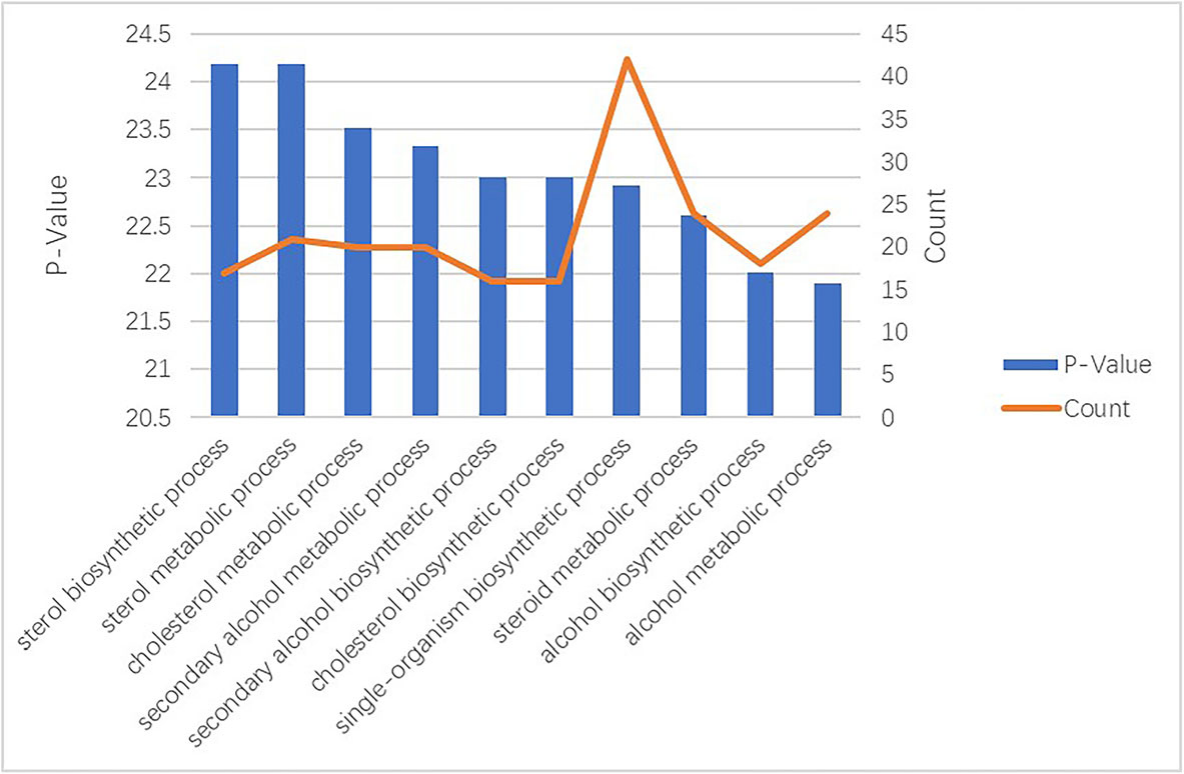
Gene Ontology enrichment analysis of hub genes

The first 10 enrichment processes of GO enrichment analysis were listed in Fig. 5. Differentially expressed genes were mainly enriched in ‘sterol biosynthetic and metabolic process’, ‘cholesterol metabolic and biosynthetic process’.

The results of KEGG pathway analysis showed all differential genes are involved in biological processes through 143 KEGG pathways. First 10 KEGG pathways were listed in Table 1.

**Table 1:** KEGG pathway enrichment analysis of DEGs between two groups

Term	Count	PValue	Input
Steroid biosynthesis	8	0.000	SQLE, TM7SF2, DHCR24, FDFT1, EBP, DHCR7, SC5D, MSMO1
Metabolic pathways	26	0.000	ME1, FDFT1, HMGCR, QPRT, IDI1, IMPA2, HPSE, EBP, DHCR24, AKR1C3, ACOX2, MVK, CSGALNACT1, FDPS, SQLE, MVD, PLPP3, ACACA, HMGCS1, MSMO1, GK, TM7SF2, SC5D, ARG2, DHCR7, SDS
Terpenoid backbone biosynthesis	6	0.000	FDPS, MVD, HMGCR, HMGCS1, MVK, IDI1
PPAR signaling pathway	7	0.000	ME1, PPARD, GK, LPL, PLIN2, ACOX2, FADS2
Hematopoietic cell lineage	5	0.000	CSF1R, CD1E, CD14, CSF1, CSF2RA
Transcriptional misregulation in cancer	6	0.000	NR4A3, CD14, NUPR1, ETV5, CSF1R, DUSP6
Cytokine-cytokine receptor interaction	7	0.000	CXCL2, CCL4, CXCR4, CCL7, CSF2RA, CSF1R, CSF1
ABC transporters	3	0.001	ABCB9, ABCG1, ABCA1
MAPK signaling pathway	6	0.002	DUSP5, CD14, GADD45A, CD14, GADD45A, DUSP6, DUSP2, PTPRR
Glycerolipid metabolism	3	0.003	GK, LPL, PLPP3

### Protein interaction network analysis

**Fig. 6:**
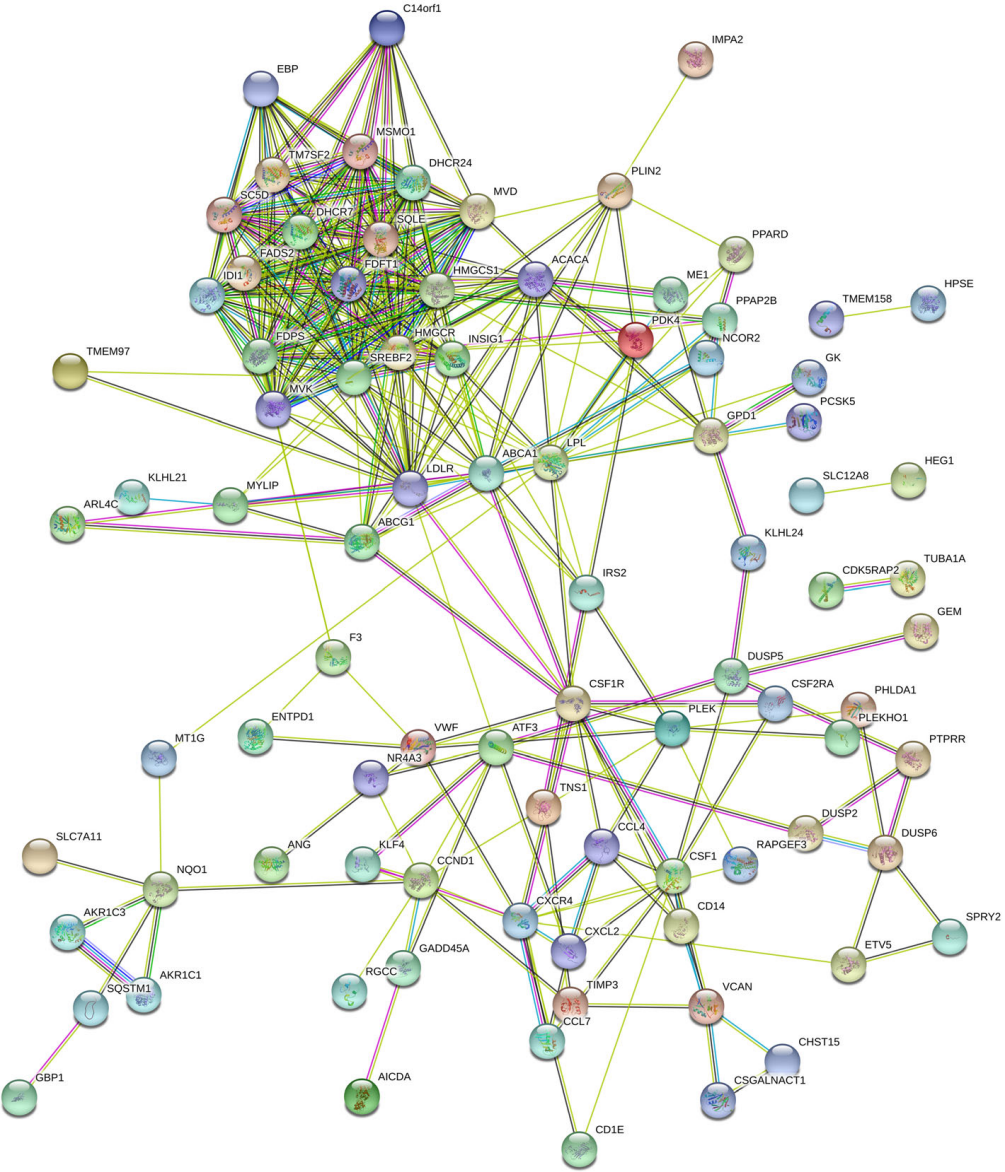
Protein–protein interaction network of differentially expressed genes

**Fig. 7:**
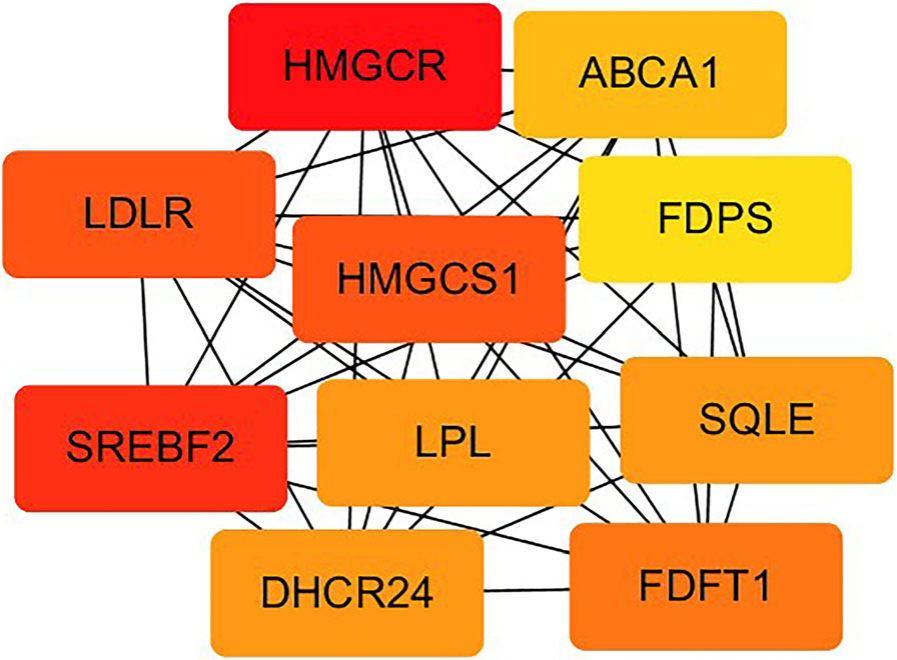
PPI network of the ten outstanding genes

A PPI network of the DEGs was constructed (Fig. 6), and 10 outstanding genes of the PPI network was identified by using Cytoscape (Fig. 7), which include HMGCR, SREBF2, HMGCS1, LDLR, FDFT1, LPL, SQLE, DHCR24, ABCA1 and FDPS.

## Discussion

The results of this study showed that the differential genes mainly regulated the transformation of macrophages into foam cells by up-regulating and down-regulating the sterol biosynthetic and metabolic process, and the cholesterol metabolic and biosynthetic process. It was mainly achieved by acting on different targets of the Steroid biosynthesis, Metabolic pathways, PPAR signaling pathway, MAPK signaling pathway, Glycerolipid metabolism, Cytokine-cytokine receptor interaction, etc. Further analysis of PPI showed that HMGCR, SREBF2, HMGCS1, LDLR, FDFT1, LPL, SQLE, DHCR24, ABCA1 and FDPS were the 10 genes that played a core role in the action network.

3-hydroxy-3-methylglutaryl CoA (HMG-CoA) is an important intermediate in cholesterol synthesis. HMGCR and HMGCS1 respectively encode two important enzymes that regulate the synthesis and further transformation of HMG-CoA. HMGCS1 encodes HMG-CoA synthase, which mediates the first step of the pathway, converting acetyl CoA and acetoacetyl-CoA into HMG-CoA. HMG-CoA is then reduced by HMG-CoA reductase (encoded by HMGCR) into mevalonate in the rate limiting step of the reaction [5]. As a rate-limiting enzyme for cholesterol synthesis, HMG-CoA reductase is the target of a variety of physiological hormones and drugs to regulate the synthesis efficiency of cholesterol [6].

The sterol-regulatory element binding transcription factors (SREBFs) has been shown to be primarily involved in cellular cholesterol homeostasis, which can regulate the expression of low-density lipoprotein (LDL) receptors by enabling the hepatocytes to remove cholesterol contained in LDL particles from the bloodstream [7]. The SREBFs comprise three SREBF isoforms, SREBF1a, SREBF-1c, and SREBF-2. SREBF-2 gene codes for SREBP-2, which is a key regulator of cholesterol. When cells are deprived of cholesterol, proteolytic cleavage releases the NH (2)-terminal domain of SREBP-2 that binds and activates the promoters of SREBP-2-regulated genes including the genes encoding the LDL receptor (LDLR), HMG-CoA synthase, and HMG-CoA reductase. Thus, SREBP-2 gene activation leads to enhanced cholesterol uptake and biosynthesis [8]. In addition, SREBF-2 variants were associated with premature CAD [9]. Seung-soon Im et al. found that SREBP-1a not only activates genes required for lipogenesis in macrophages but also the gene encoding Nlrp1a, which is a core inflammasome component [10]; SREBP2 with SREBP cleavage activating protein (SREBP cleavage activating protein, SCAP) formed SCAP-SREBP2 complex, which was required for optimal activation of the NLRP3 inflammasome both in vitro and in vivo [11], promote local inflammatory response in arterial wall.

LDLR is an integral membrane protein which is most abundantly expressed in the liver, and binds to and removes LDL-C from the circulation by endocytosis [12, 13]. SREBP-2 can negatively regulate the expression level of LDLR gene at the transcriptional level [14]. Additionally, post-translational regulation of LDLR is primarily governed by PCSK9 [15], and the post-transcriptional regulation of LDLR is mainly achieved through modulation of its mRNA stability [16].

Farnesyl diphosphate farnesyl transferase 1 (FDFT1) encoded squalene synthase, which is another key enzyme for the synthesis of sterols, and ultimately cholesterol [17]. The human FDFT1 gene spans over 40 kb on chromosome 8p.23 [18], which is ubiquitously expressed in human tissues but is particularly high in the hypothalamus and liver [19, 20]. The FDFT1 gene has several isoforms, with the most common containing eight exons. The promoter of the gene contains three SRE-like sequences (SRE-1, Inv-SRE-3 and SRE-1), which are located between 198 and 127 bp upstream of the predominant transcription start site [21, 22]. SREBPs bind to SRE-like sequences to regulate transcription of the FDFT1 gene [21, 23].

Lipoprotein lipase (LPL) encoded by the LPL gene hydrolyses triglycerides in circulating chylomicrons, LDL and very low-density lipoproteins (VLDL) to render non-unesterified fatty acids (NEFA) and 2-monoacylglycerol for tissue utilization [24]. The catalytic activity of plasma LPL can reduce plasma TG level and increase HDL-C level, and thus appears to be antiatherogenic [25]. And studies have confirmed that activation of peroxidase activated value-added receptor (PPAR) on the nuclear membrane increases the gene expression of LPL [26, 27], which is a target for drugs that lower triglycerides. On the other hand, noncatalytic activity of lipoprotein enzymes can enhance atherosclerosis through bridging and selective uptake of CE [28], but the mechanism is more complex.

3β-hydroxysterol Δ24-reductase (DHCR24) encodes the cholesterol-synthesizing enzyme seladin-1, and catalyzes the final step of Bloch cholesterol synthetic pathway [29]. Like many cholesterol synthetic genes, DHCR24 is transcriptionally regulated by sterols via SREBF [30]. And, due to its critical role in cholesterol synthesis, DHCR24 is a prime candidate that acts as a control point for regulation of cholesterol besides HMG-CoA reductase [31]. In addition, independent of cholesterol metabolism, Fei Han et al. found that DHCR24 attenuate cardiac infarction and dysfunction by anti-apoptotic effect [32].

Squalene epoxidase (SQLE) encodes a monooxygenase, which is the second rate-limiting enzyme in cholesterol biosynthesis by catalyzing the first oxygenation step in sterol biosynthesis [33]. SQLE exertncvhvk n s this effect through the action of two key downstream metabolites, cholesteryl ester and nicotinamide adenine dinucleotide phosphate (NADP+) [34]. Additionally, SQLE is also a target of the SREBP-2. It has been found that there were two transcription factors of SP1 and NF–Y in SQLE [35].

Efflux of cholesterol is accompanied by cholesterol transport proteins including adipocyte ATP-binding cassette A1 (ABCA1), adipocyte ATP-binding cassette G1 (ABCG1) and class B scavenger receptor (SR-BI) [36]. It is now well established that ABCA1 plays a critical role in the prevention of macrophage foam cell formation and atherosclerosis by mediating the active transport of intracellular cholesterol and phospholipids to apoA-I, the major lipoprotein in HDL [37].

Farnesyl diphosphate synthase (FDPS) is a branch point enzyme in the synthesis of sterols and isoprenylated cellular metabolites. FDPS catalyzes the conversion of isopentenyl pyrophosphate and dimethylallyl pyrophosphate to geranyl pyrophosphate and farnesyl pyrophosphate, which are protein prenylation substrates [38]. FDPS is mainly known to mediate immunoregulatory functions [39, 40], its activity and expression have been also documented in human colon cancer [41] and certain other neoplastic disorders. Therefore, it may be a potential target for cancer treatment.

In this study, it was found that the interaction network with HMGCR and SREBF-2 et al. as the core was involved in the formation of foam cells. This is not only the mechanism leading to the formation of atherosclerosis, but also the further induction of local inflammatory response in the vascular wall is another major cause of the formation of AS. But it is still to be further studied to clarify which one comes first or even which one is more important. The cause-and-effect relationship between lipid deposition and inflammatory response, as well as its core link, needs to be further studied and clarified.

## Conclusion

In this study, it was found that lipid metabolism related genes and molecular pathways were the key to the transformation of macrophages into foam cells. Currently widely used and effective lipid-lowering drugs can not only reduce lipid levels, but also further reduce ASCVD risk through lipid-lowering. Although the mechanism of action has been partially clarified, the results of this study further suggest that the ability of monocytes to differentiate into macrophages and further turn into foam cells in the population may be related to premature CAD, and the mechanism may be mainly related to genes involved in lipid metabolism.

## Data Availability

The gene expression profiles of GSE9874 were downloaded from Gene Expression Omnibus (GEO) (https://www.ncbi.nlm.nih.gov/geo/query/acc.cgi?acc=GSE9874).

## Abbreviations

AS: Atherosclerosis
ABCA1: ATP-binding cassette A1
ABCG1: ATP-binding cassette G1
SR-BI: Class B scavenger receptor
CAD: Coronary artery disease
DEGs: Differentially expression genes
DHCR24: 3β-hydroxysterol Δ24-reductase
FDFT1: Farnesyl diphosphate farnesyl transferase 1
FDPS: Farnesyl diphosphate synthase
GEO: Gene expression omnibus
GO: Gene ontology
HMG-CoA: 3-hydroxy-3-methylglutaryl CoA
LDL: Low-density lipoprotein
LDLR: LDL receptor
LPL: Lipoprotein lipase
NADP+: Nicotinamide adenine dinucleotide phosphate
NEFA: Non-unesterified fatty acids
PPAR: Peroxidase activated value-added receptor
PCA: Principal component analysis
SREBFs: Sterol-regulatory element binding transcription factors
SQLE: Squalene epoxidase
VLDL: Very low-density lipoproteins
